# Web surveys during the COVID-19 pandemic: a critical look for collective learning

**DOI:** 10.1590/0102-311XEN174523

**Published:** 2025-07-04

**Authors:** Martha Cristina Nunes Moreira, Dimitri Marques Abramov, Karla Gonçalves Camacho, Maria de Fátima Junqueira-Marinho, Adriana Teixeira Reis, Saint Clair dos Santos Gomes, Daniella Campelo Batalha Cox Moore

**Affiliations:** 1 Instituto Nacional de Saúde da Mulher, Criança e Adolescente Fernandes Figueira, Fundação Oswaldo Cruz, Rio de Janeiro, Brasil.; 2 Universidade do Estado do Rio de Janeiro, Rio de Janeiro, Brasil.; 3 Universidade Federal Fluminense, Niterói, Brasil.

Web surveys is a methodology that speeds up the collection and dissemination of data and information, and has the potential to reach a large audience in a short period of time with a relatively low use of resources compared to traditional methods, putting them at an advantage when the answers in science require speed ^1^ ^,^ ^2^. The COVID-19 pandemic has boosted web surveys since the online data collection method respected social distancing and ideally enhanced the participants’ available time to fill out survey forms. Recognizing its characteristics, this article brings reflections based on the authors’ experience in web surveys conducted to assess vaccine hesitancy, the impact of the pandemic on health professionals, the opinion of Brazilians about the lockdown and the return to in-person classes in schools for its use and improvement in future work.

Web survey planning must consider the diversity of the participating groups and the risks and benefits of a certain portion not being adequately represented. Its limits stand out when researchers propose solutions understanding groups as a homogeneous entity which can, depending on the subsequent use of the results, further aggravate existing inequalities. According to Cetic.br data the number of Brazilian households with access to information and communication technologies (ICT) increased from 13% to 85% in 20 years ^3^. However, connectivity is plagued with inequalities that compromise and hinder the construction of traditional sampling plans, with group weightings and representativeness. According to the survey, 73% of class A households have satisfactory connectivity against 3% of class D/E; 33% of South households against 11% of Northeast; and 28% of male-headed households against 16% of female-headed. Hardly a survey or any fully digital approach will be able to reach all strata of the population. We must therefore recognize that as the digital environment, especially in health care, operates as a “super” social determinant ^4^ the existing inequalities in access to digital devices and digital literacy skills compromise digital equity, the health and well-being of the population.

Wilson et al. ^5^ found that investment in user-friendly designs, software interfaces and websites with culturally appropriate navigability and content, along with the provision of support infrastructure for users, including devices, free connectivity and non-digital options, can help increase participation. The authors valued the educational support of family members, friends or professionals to aid individuals in developing their digital literacy skills and their use in the context of health care in digital environments. Still on the subject of representativeness, another criticism of web surveys comes from the response rate to ensure survey reliability. In Brazil, with its great diversity and plurality, we must reflect on what can be considered a representative result in the use of web surveys. Access to technology and the response rate of a locality or country can even vary according to its development index ^6^. As such, web survey planning must consider the inequalities in ICTs access, in the time available and in the literacy of low-income people. Added to this is the need to generate interest and trust, using accessible language alongside the ethical implications and costs involved.

This scenario was repeated in the numerous web surveys conducted: most participants were female with high schooling level and income, results which highlight the need for a critical look at the use of web surveys to generalize sample data to the general population and selection bias ^7^ ^,^ ^8^. It is also worth discussing trust, engagement and the feeling of being a co-participant in science. Classical economic theory suggests that people make a rational choice to take part in research after weighing up the costs and benefits. Leverage-saliency theory proposes a variety of factors that interfere with the decision to participate in research: financial motivation, social exchange and interest in the study subject. This theory suggests understanding, prior to conducting the study, which motivations could generate greater participation in each group. Longworth et al. ^9^ identified that engagement in web surveys is related to participants’ perception of feeling listened to and believing that the intervention is being planned to respond to the real needs of the communities. Another strategy to improve the analysis of these under-represented groups is weighting the sample. But this must be conducted with planning - even though it has the potential to correct discrepancies in many situations, in some cases its effect is nil or even unpredictable depending on the set of variables analyzed ^10^.

We experienced many of these questions and dilemmas in the web surveys conducted during the pandemic, which had a good response rate. A large part of the motivation to take part, we believe, is the strong presence of the issues addressed on the internet and the whole political health moment. In the case of these surveys, different communication strategies were used to present the same form on social media throughout the data collection period. We tried to use simple language on the form, incorporating expressions, beliefs and thoughts that were prevalent on social media at the time. We also created an account on a popular photo and video sharing social network to interact with the public and reformulate the questions, the so-called web-push methodology ^2^ ^,^ ^8^ ^,^ ^10^.

The above example serves as a bridge for discussing how to attract and recruit participants in a web survey that can be disseminated in the universe of social networks, which often involves the researchers’ own networks of friendship, work and activism, and can thus generate a specific profile of respondents based on class, education, race and gender. Research craftsmanship can gain from the participation of groups that have community capillarity and diversity, promoting interest, mobilization and collaborative knowledge construction. These collaborative ties have to precede health emergencies, as they need time to build trust and credibility. Even so, underprivileged population groups or those excluded from social rights as well as digital access tend to be underrepresented ^10^.

We believe that the inclusion of disadvantaged groups in research that affects their health, as observed in the COVID-19 pandemic, from team formation to the discussion and dissemination of the results, can contribute to citizen science which is characterized by co-creation, with a team made up of researchers and citizens, attentive to the factors that influence public health for diverse groups ^9^. This perspective can increase its dissemination and the involvement of participants from the lower classes and urban periphery groups. Such approach can help to measure the weight of the difficulty in accessing internet or technological devices and the legitimate lack of interest in participating in health research, which can arise from historical exclusion in decision-making processes that affect trust. Additionally, strategies such as the use of shorter self-administered questionnaires can reduce fatigue by minimizing interlocutor absence with simple, close language and accessibility resources for people with visual, intellectual and hearing disabilities. By including people with disabilities in the research team who have knowledge and techniques in communication accessibility, the co-creation process can increase inclusion, awareness and dissemination of science. On the other hand, concepts like co-design, co-production and co-creation, especially in low- and middle-income countries, can foster greater engagement and favor results aligned with users’ needs, improving the formulation of public policies.

Finally, the use of web surveys, so prolific during the pandemic, deserves to be analyzed in light of the above discussions before the next public health emergency, so that greater benefits can come from this research design in the future.
