# Supplementary material for: Web surveys during the COVID-19 pandemic: a critical look for collective learning
Source: Cad Saude Publica. 2025 Jul 4;41(6):e00174523. doi: 10.1590/0102-311XEN174523 (PMC12236086; doi:10.1590/0102-311XEN174523)
Supplement: Translation

# Translation: *Web surveys* na pandemia de COVID-19: um olhar crítico para aprendizados coletivos

**DOI:** 10.1590/0102-311XPT174523

**Authors:** Martha Cristina Nunes Moreira, Dimitri Marques Abramov, Karla Gonçalves Camacho, Maria de Fátima Junqueira-Marinho, Adriana Teixeira Reis, Saint Clair dos Santos Gomes, Daniella Campelo Batalha Cox Moore

**Affiliations:** 1 Instituto Nacional de Saúde da Mulher, Criança e Adolescente Fernandes Figueira, Fundação Oswaldo Cruz, Rio de Janeiro, Brasil.; 2 Universidade do Estado do Rio de Janeiro, Rio de Janeiro, Brasil.; 3 Universidade Federal Fluminense, Niterói, Brasil.

Os *web surveys* representam uma metodologia que agiliza a obtenção e divulgação de dados e informações. Têm potencial para alcançar um grande número de participantes em um curto período de tempo, com um uso de recursos relativamente baixo quando comparados aos métodos tradicionais, os colocando em vantagem quando as respostas para a ciência precisam ser ágeis ^1^ ^,^ ^2^. A pandemia de COVID-19 impulsionou os *web surveys*, uma vez que o método de coleta de dados online respeitava o distanciamento social e potencializava, idealmente, o tempo disponível dos participantes para preencher formulários de pesquisa. Desta forma, reconhecendo suas características, neste artigo são feitas reflexões, a partir da vivência dos próprios autores em *web survey* conduzidos para avaliação da hesitação vacinal, do impacto da pandemia sobre os profissionais de saúde, da opinião dos brasileiros sobre o *lockdown* e do retorno ao modelo presencial nas escolas, para seu uso e aprimoramento em trabalhos futuros.

O planejamento de *web surveys* deve considerar a diversidade dos grupos participantes e ponderar desde a concepção sobre os riscos e benefícios de determinada parcela não ser representada adequadamente. Seus limites são destacados quando os pesquisadores produzem soluções entendendo grupos como uma entidade homogênea, o que pode, a depender do uso posterior dos resultados, agravar ainda mais as desigualdades existentes. Segundo dados do Cetic.br, houve uma ampliação de domicílios brasileiros com acesso às tecnologias da informação e comunicação (TIC), passando de 13% para 85% em 20 anos ^3^. No entanto, com relação à conectividade, são observadas desigualdades que comprometem e dificultam a construção de planos amostrais tradicionais, com ponderações e representatividade dos grupos. Pelo levantamento, com conectividade satisfatória encontram-se 73% dos domicílios da classe A, contra 3% da classe D/E; 33% da Região Sul, contra 11% do Nordeste; e 28% dos homens, contra 16% das mulheres. Dificilmente um *survey* ou qualquer abordagem totalmente digital conseguirá alcançar todos os estratos da população. Desta forma, é necessário reconhecer que, mesmo o ambiente digital, principalmente no cuidado à saúde, operando como um “super” determinante social ^4^, as desigualdades no acesso aos dispositivos digitais e habilidades no seu uso comprometem a equidade digital, a saúde e o bem-estar da população.

Estudo realizado por Wilson et al. ^5^ avaliou que o investimento em *designs* amigáveis ao usuário, interfaces de softwares e sites com navegabilidade e conteúdo culturalmente apropriados, juntamente com o oferecimento de infraestrutura de suporte aos usuários, incluindo dispositivos, conectividade gratuita e opções não digitais, pode contribuir para incrementar a participação. Os autores valorizaram o suporte educacional de familiares, amigos ou profissionais para apoiar os indivíduos a desenvolverem suas habilidades de alfabetização digital e seu uso no contexto da atenção à saúde em ambientes digitais. Ainda no tema da representatividade, outra crítica aos *web surveys* advém da taxa de respostas para garantir a confiabilidade da pesquisa. Na realidade brasileira, com uma grande diversidade e pluralidade, é necessário refletir, de fato, o que se pode considerar como um resultado representativo no uso de *web surveys*. O acesso à tecnologia e a taxa de resposta de uma localidade ou país pode, inclusive, variar, de acordo com seu índice de desenvolvimento ^6^. Desta forma, o planejamento de um *web survey* deve considerar as inequidades no acesso às TIC, as desigualdades no tempo disponível e no letramento para preenchimento por parte de pessoas pobres e periféricas. A isso se reúne a necessidade de gerar interesse e confiança, com recursos de uma linguagem acessível, *pari passu* às implicações éticas e custos envolvidos.

Esse cenário se repetiu nos inúmeros *web surveys* realizados que se caracterizaram por uma participação majoritária de um grupo com maior escolaridade, maior renda e do sexo feminino, como resultado das análises, remete ao que foi problematizado até aqui, quando se reforçou a necessidade de um olhar crítico sobre o uso de *web surveys* para generalização dos dados da amostra para a população e o viés de seleção ^7^ ^,^ ^8^. Além disto, vale discutir a confiança, o engajamento e o sentimento de ser um coparticipante da ciência. A teoria econômica clássica sugere que as pessoas fazem uma escolha racional para participar de uma pesquisa após pesar seus custos e benefícios. A *leverage-saliency theory* propõe uma variedade de fatores que interferem na decisão de participação em uma pesquisa: a motivação financeira, a troca social e o interesse pelo tema de estudo. Esta teoria sugere compreender, previamente à realização do estudo, quais motivações poderiam gerar maior participação em cada grupo. Em um estudo realizado por Longworth et al. ^9^ foi possível identificar que o engajamento nos *web surveys* está relacionado com a percepção do participante de sentir-se escutado e de acreditar que a intervenção está sendo planejada para responder às reais necessidades das comunidades. Outra estratégia para melhorar a análise desses grupos sub-representados seria a ponderação da amostra. Entretanto, esta deve ser executada com planejamento, pois, mesmo tendo potencial para corrigir discrepâncias em muitas situações, existem casos em que seu efeito é nulo ou até mesmo imprevisíveis dependendo do conjunto de variáveis analisadas ^10^.

Os autores experimentaram boa parte destas questões e dilemas nos *web surveys* conduzidos ao longo da pandemia e que tiveram uma boa taxa de resposta. Acredita-se que boa parte da motivação em participar seja a forte presença das temáticas abordadas na internet e todo o momento político sanitário vivenciado. No caso destas pesquisas, foram utilizadas diferentes estratégias de comunicação para apresentar o mesmo formulário nas redes sociais ao longo do tempo de coleta de dados. Buscou-se utilizar uma linguagem simples no formulário, com a incorporação de expressões, crenças e pensamentos que predominavam nas redes sociais naquele momento. Além disto, foi criada uma conta numa rede social de compartilhamento de fotos e vídeos de grande popularidade para a interação com o público e reformulação das questões, a chamada metodologia *web-push* ^2^ ^,^ ^8^ ^,^ ^10^.

O exemplo anterior serve de ponte para que se possa discutir a forma de captação e recrutamento dos participantes em um *web survey*, os quais podem ser disseminados no universo das redes sociais, que muitas vezes passa pelas redes de relações de amizade, trabalho, ativismo dos próprios pesquisadores, o que pode gerar um perfil específico de respondentes em recortes de classe, escolaridade, raça e gênero. O artesanato das pesquisas pode ganhar com a participação de grupos que tenham capilaridade comunitária e diversidade, promovendo interesse, mobilização e construção colaborativa de conhecimento. Esses vínculos de colaboração têm que preceder emergências sanitárias, pois precisam de tempo para construção de confiança e credibilidade. Ainda assim, grupos populacionais menos favorecidos ou excluídos de direitos sociais como, também, de acesso digital, tendem a estar sub-representados ^10^.

Acredita-se que a inserção de grupos menos favorecidos em pesquisas que afetam sua saúde, como observado na pandemia de COVID-19, desde a formação da equipe até a discussão e disseminação dos resultados, possa contribuir com uma ciência cidadã, a qual se caracteriza pela cocriação, com uma equipe composta por pesquisadores e cidadãos, atenta aos fatores que influenciam a saúde pública para grupos diversos ^9^. Essa perspectiva pode incrementar sua disseminação e a vinculação dos participantes de classes populares e grupos da periferia urbana. Este artesanato pode ajudar a dimensionar o peso da dificuldade de acesso à internet ou dispositivos tecnológicos e falta legítima de interesse em participar de pesquisas em saúde, que pode surgir da exclusão histórica nos processos decisórios que afetam a confiança. Além disto, estratégias como o uso de questionários autoaplicados mais curtos, podem reduzir a fadiga, minimizando a ausência do interlocutor, com linguagem simples, próxima e com recursos de acessibilidade para pessoas com deficiência visual, intelectual e auditiva. Ao contemplar na equipe de pesquisa pessoas com deficiência com conhecimentos e técnicas em acessibilidade comunicacional, o processo de co-criação pode incrementar a inclusão, sensibilização e disseminação da ciência. Já conceitos como o codesign, coprodução e cocriação, especialmente em países de baixa e média renda, podem propiciar um maior engajamento e favorecer resultados alinhados às necessidades dos usuários, aprimorando a formulação de políticas públicas.

Por fim, o uso de *web surveys*, tão exuberante durante a pandemia, merece ser analisado à luz das discussões que trouxemos aqui, antes da próxima emergência de saúde pública, para que maiores benefícios possam advir deste desenho de pesquisa no futuro.
